# Virulence of invasive *Salmonella* Typhimurium ST313 in animal models of infection

**DOI:** 10.1371/journal.pntd.0005697

**Published:** 2017-08-04

**Authors:** Girish Ramachandran, Aruna Panda, Ellen E. Higginson, Eugene Ateh, Michael M. Lipsky, Sunil Sen, Courtney A. Matson, Jasnehta Permala-Booth, Louis J. DeTolla, Sharon M. Tennant

**Affiliations:** 1 Center for Vaccine Development and Institute for Global Health, University of Maryland School of Medicine, Baltimore, Maryland, United States of America; 2 Department of Medicine, University of Maryland School of Medicine, Baltimore, Maryland, United States of America; 3 Department of Pathology, Program of Comparative Medicine, University of Maryland School of Medicine, Baltimore, Maryland, United States of America; 4 Department of Epidemiology and Public Health, University of Maryland School of Medicine, Baltimore, Maryland, United States of America; 5 Institute of Human Virology, University of Maryland School of Medicine, Baltimore, Maryland, United States of America; Institut Pasteur, FRANCE

## Abstract

*Salmonella* Typhimurium sequence type (ST) 313 produces septicemia in infants in sub-Saharan Africa. Although there are known genetic and phenotypic differences between ST313 strains and gastroenteritis-associated ST19 strains, conflicting data about the *in vivo* virulence of ST313 strains have been reported. To resolve these differences, we tested clinical *Salmonella* Typhimurium ST313 and ST19 strains in murine and rhesus macaque infection models. The 50% lethal dose (LD_50_) was determined for three *Salmonella* Typhimurium ST19 and ST313 strains in mice. For dissemination studies, bacterial burden in organs was determined at various time-points post-challenge. Indian rhesus macaques were infected with one ST19 and one ST313 strain. Animals were monitored for clinical signs and bacterial burden and pathology were determined. The LD_50_ values for ST19 and ST313 infected mice were not significantly different. However, ST313-infected BALB/c mice had significantly higher bacterial numbers in blood at 24 h than ST19-infected mice. ST19-infected rhesus macaques exhibited moderate-to-severe diarrhea while ST313-infected monkeys showed no-to-mild diarrhea. ST19-infected monkeys had higher bacterial burden and increased inflammation in tissues. Our data suggest that *Salmonella* Typhimurium ST313 invasiveness may be investigated using mice. The non-human primate results are consistent with clinical data, suggesting that ST313 strains do not cause diarrhea.

## Author summary

*Salmonella* Typhimurium sequence type (ST) 19 causes self-limiting gastroenteritis throughout the world. In recent years, a new ST of *Salmonella* Typhimurium, ST313, has been circulating in sub-Saharan Africa and causes fever in infants and immunocompromised individuals in the absence of gastroenteritis. The differences in pathogenesis between *Salmonella* Typhimurium ST313 and ST19 is not clearly understood in animal models. In our current study, we examined possible differences between ST19 and ST313 strains in different animal models. We found that ST313 strains could replicate in the blood of mice to higher levels than ST19 strains. Furthermore, when rhesus macaques were orally infected with *Salmonella* Typhimurium ST19 or ST313, we observed increased diarrhea, inflammation and higher bacterial burden in ST19-infected monkeys compared to ST313-infected monkeys. Our data supports the clinical evidence which suggests that *Salmonella* Typhimurium ST313 do not cause gastroenteritis and instead are more invasive than ST19 strains.

## Introduction

*Salmonella enterica* serovar Typhimurium generally causes gastroenteritis in immunocompetent individuals. However, there is growing evidence to suggest that in certain susceptible populations, such as infants in sub-Saharan Africa and HIV-positive adults, *Salmonella* Typhimurium infection manifests as septicemia without any gastroenteritis [1,2,3].

There is a novel genotype of *Salmonella* Typhimurium circulating in Africa called sequence type (ST) 313 (based on multi-locus sequence typing) [4]. In contrast, the most common ST present throughout the rest of the world is ST19 [5]. Kingsley et al [4] compared the genome of an ST313 strain, D23580, from Malawi, with the genomes of *Salmonella* Typhimurium ST19 gastroenteritis-associated strains and observed that D23580 appeared to have undergone genome degradation similar to what has been observed for *Salmonella* Typhi and Paratyphi A [6]. Okoro et al [7] further describe two lineages of invasive *Salmonella* Typhimurium, lineage I and lineage II, which emerged at the same time as HIV in Africa. The authors hypothesize that lineage I isolates have been replaced by lineage II isolates due to the use of chloramphenicol for treatment of invasive non-typhoidal *Salmonella* (iNTS).

Phenotypic analysis has also shown that *Salmonella* Typhimurium ST313 strains differ from ST19 strains. Two groups determined that ST313 isolates produce less flagella and are less pro-inflammatory than ST19 isolates [8,9]. We previously showed that ST313 strains are less motile than ST19 [9] whereas Yang et al [10] observed that D23580 (ST313) is just as, if not more, motile than *Salmonella* Typhimurium SL1344 (ST19). *In vitro*, Ramachandran et al [9] showed that invasive ST313 isolates from Mali survive well within macrophages and cause less host cell death than clinical ST19 isolates.

The *in vivo* infection data for *Salmonella* Typhimurium ST313 has been mixed, with several groups showing conflicting data. ST313 strains have been shown to colonize systemic sites of C57BL/6 and BALB/c mice [10,11]. Yang et al [10] found that D23580 (ST313) was able to more rapidly colonize the spleen than SL1344 (ST19). Similarly, D23580 caused severe invasive infection in chickens and rapidly infected the spleen and liver by day 3 post-infection (p.i.) [12]. In chickens, ST313 was less able to colonize the gastrointestinal tract than ST19 [12]. Several groups have attempted to determine whether ST313 strains are able to elicit inflammation and gastroenteritis. Okoro et al [11] tested the ability of ST313 isolates to induce an inflammatory response in streptomycin-treated C57BL/6 mice and found no significant difference in colonization of the cecum but saw reduced inflammation compared to SL1344 (ST19). In contrast, Singletary et al [13] observed no significant difference in intestinal pathology of streptomycin-treated CBA/J mice infected with either D23580 (ST313) or IR715 (ST19). Fluid accumulation and inflammation of ST313 has also been evaluated in ileal loop models. In bovine ileal loops, ST313 strains induced significantly less fluid accumulation than ST19 isolates [11]. In a rhesus macaque ileal loop model, no difference was observed between D23580 (ST313) and IR715 (ST19) in terms of fluid accumulation or inflammatory cytokine expression [13].

In this study, our goal was to evaluate virulence of *Salmonella* Typhimurium ST313 strains isolated from the blood of infants in Mali in various animal models. In particular, we sought to confirm whether previous findings from other groups are robust and to definitively assess whether ST313 can produce gastroenteritis in a rhesus macaque infection model. This would provide critical evidence to support the clinical data which suggests that ST313 strains do not produce diarrhea in infants and children.

## Materials and methods

### Ethics statement

All animal experiments were carried out in strict accordance with the recommendations in the Guide for the Care and Use of Laboratory Animals of the National Institutes of Health. All protocols were reviewed and approved by the Animal Care and Use Committee at the University of Maryland, School of Medicine. The IACUC protocol numbers for the mouse experiments were #0715010 and #0212016 and for the rhesus macaque experiment the protocol number was #1115011. Details of primate welfare, including information about housing, feeding and environmental enrichment, pain relief and method of euthanasia are described in S1 Text. The anonymized invasive NTS isolates from Mali were previously collected under a clinical protocol reviewed by the Ethics Committee of the Faculté de Medécine de Pharmacie et d'Odontostomatologie, University of Mali, and by the Institutional Review Board of the University of Maryland, Baltimore as described [14].

### Bacterial strains and culture conditions

Invasive *Salmonella* Typhimurium ST313 (D65, Q55, S11) and *Salmonella* Typhimurium ST19 (I77, I41, S52) have previously been isolated from the blood of infants in Mali, West Africa [14,15,16]. These clinical strains were maintained on animal product-free Hy-Soy (HS) agar (0.5% Hy-yest [Kerry Biosciences, Beloit, WI], 1% Soytone [TEKNova, Hollister, CA], 0.5% NaCl [American Bio, Natick, MA]).

### Mouse experiments

#### Animals

BALB/c and CD-1 outbred mice were purchased from Charles River Laboratories (Wilmington, MA).

#### 50% lethal dose (LD_50_) of *Salmonella* Typhimurium ST313 and ST19 strains in BALB/c and CD-1 mice

Ten-fold dilutions (10^3^–10^7^ CFU; n = 3) of *Salmonella* Typhimurium ST19 strains (I77, I41, S52) and ST313 strains (D65, Q55, S11) were administered by oral gavage, to 4-week-old and 8-week-old female BALB/c mice and intraperitoneally (i.p.) to 8-week-old CD-1 mice. Infected mice were weighed and monitored daily for 28 days. The 50% lethal dose of each strain was calculated by linear regression analysis.

#### Bacterial load in organs and blood

Eight-week-old female CD-1 mice (n = 3) were infected with 1 x 10^9^ CFU of *Salmonella* Typhimurium ST313 strains (D65, Q55, and S11) and *Salmonella* Typhimurium ST19 strains (I77, I41, and S52) by gavage. Mice were euthanized at 3, 24, 72 and 168 h p.i. and blood, spleen and liver were collected. Bacterial loads were determined by performing viable counts. The experiment was repeated with eight-week-old BALB/c mice (n = 5) infected by gavage with 1 x 10^9^ CFU of *Salmonella* Typhimurium D65 (ST313) and I77 (ST19). Bacterial loads were determined at 3 h and 24 h p.i.

### Rhesus macaque experiments

#### Animals

Indian-origin rhesus macaques (*Macaca mulatta*; 2 to 3 years old; males) were purchased from Alpha Genesis (Yamassee, SC). All animals were negative for anti-*Salmonella* LPS serum IgG antibodies.

#### Infection

Rhesus macaques (n = 3) were infected intragastrically with 3 x 10^9^ CFU of either *Salmonella* Typhimurium I77 (ST19; animals DFK0, DFP4, DG15) or D65 (ST313; animals DFN1, DFT9, DFV4), suspended in 8.4% sodium bicarbonate solution (Neogen Vet, Lexington, KY). Monkeys were weighed on Days 0, 1, 3, 7, 15 and 21 or 22 p.i., and blood was collected to determine chemistry, cell counts and culture (ANTECH Diagnostics, Lake Success, NY), and cytokine responses (as described in S1 Text). Fresh fecal samples were collected on Days 0 to 7, 9, 11, 15, 18 and 21 or 22 to determine fecal shedding. *Salmonella* Typhimurium was quantified by serially diluting 1 g of feces 10-fold in saline. The samples were cultured on *Salmonella*-Shigella (SS) Agar plates (BD, Sparks, MD) and CFUs were determined. The monkeys were monitored daily to observe for clinical signs of diarrhea, lethargy, dysentery, weight loss and fever. At necropsy on days 21 or 22, organs were swabbed for bacterial culture (ANTECH Diagnostics). Spleen, liver, ileum, colon and mesenteric lymph node (MLN) samples were homogenized using a tissue homogenizer (Omni International, Kennesaw, GA). Serial dilutions of the samples were plated onto SS Agar plates to determine the bacterial loads. Tissues collected at necropsy were formalin fixed, paraffin-embedded and stained with Haematoxylin Eosin (H&E) to determine gross inflammation, polymorphonuclear leukocyte (PMN) infiltration, and epithelial barrier integrity. Slides were examined in a blinded manner by a veterinary pathologist.

### Statistical analysis

Data were generally analysed by using Student’s unpaired t-test or Mann-Whitney test, two-tailed. Analysis of diarrheal episodes was carried out using the Mann-Whitney test, one-tailed, and Fisher’s exact test, one-tailed. Statistical analysis was completed using Prism 5 (GraphPad software, Inc, La Jolla, CA).

## Results

### Comparable 50% lethal dose (LD_50_) for *Salmonella* Typhimurium ST19 and ST313 in CD-1 and BALB/c mice

No significant difference in LD_50_ was observed for ST313 versus ST19 strains. Four-week-old (juvenile) BALB/c mice were challenged perorally (p.o.) with three strains of *Salmonella* Typhimurium ST19 (I77, I41, S52) and ST313 (D65, Q55, S11). The oral LD_50_ values were highly variable between strains, but showed no clear trend between sequence types (Table 1). When eight-week-old BALB/c mice were infected p.o., the geometric mean oral LD_50_ for the ST19 strains was determined to be 1.2 x 10^4^ CFU and for the ST313 strains, 5.0 x 10^4^ CFU. Similarly, in CD-1 mice, the i.p. LD_50_ was 1.1 x 10^4^ CFU and 5.1 x 10^4^ CFU for the ST19 and ST313 strains, respectively.

**Table 1:** 50% lethal dose of ST19 and ST313 strains in juvenile BALB/c and adult BALB/c and CD-1 mice.

Age	Mouse strain	Infection route	Sequence type	Bacterial strain	LD_50_ (CFU)	Geometric mean ± SD LD_50_ (CFU)	P value (Student’s t-test)
Juvenile mice (4-week-old)	BALB/c	p.o.	ST19	I77	3.0 x 10^4^	ND	ND
				I41	6.5 x 10^5^		
				S52	2.3 x 10^2^		
			ST313	D65	3.9 x 10^4^	ND	
				Q55	7.4 x 10^1^		
				S11	1.3 x 10^2^		
Adult mice (6–8 week-old)	BALB/c	p.o.	ST19	I77	2.0 x 10^4^	1.19 x 10^4^ ± 1.0 x 10^4^	0.12
				I41	2.2 x 10^4^		
				S52	3.8 x 10^3^		
			ST313	D65	2.0 x 10^4^	5.0 x 10^4^ ± 3.4 x 10^4^	
				Q55	7.9 x 10^4^		
				S11	7.9 x 10^4^		
	CD-1	i.p.	ST19	I77	1.23 x 10^5^	1.13 x 10^4^ ± 6.9 x 10^4^	0.40
				I41	2.0 x 10^3^		
				S52	5.8 x 10^3^		
			ST313	D65	6.3 x 10^3^	5.14 x 10^4^ ± 8.8 x 10^4^	
				Q55	1.8 x 10^5^		
				S11	1.2 x 10^5^		

ND, Not determined

### *Salmonella* Typhimurium ST313 shows higher levels of bacteremia compared to ST19 strains

**Fig 1:**
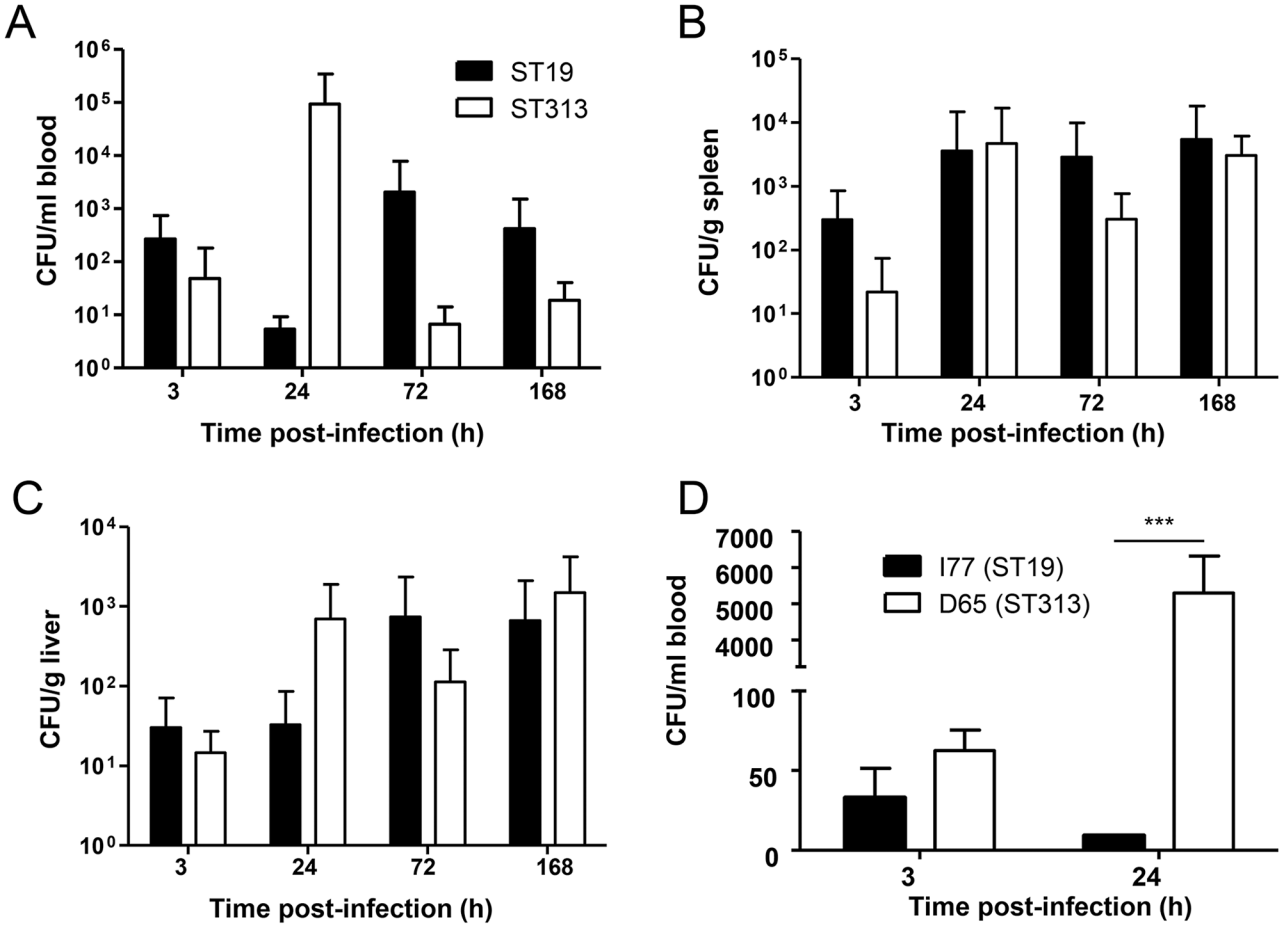
Bacterial load of *Salmonella* Typhimurium ST313 and ST19 in mice. CD-1 mice were infected perorally with 3 strains of *Salmonella* Typhimurium ST19 (I77, I41, S52) and ST313 (D65, Q55, S11). The number of CFUs at 3, 24, 72 and 168 h p.i. were determined in blood (A), spleen (B) and liver (C). BALB/c mice were infected perorally with *Salmonella* Typhimurium I77 and D65 and blood harvested at 3 and 24 h p.i. (D). Results are expressed as mean ± SD. *** represents P < 0.001, Student’s t-test, two-tailed.

We next investigated the infection kinetics of *Salmonella* Typhimurium ST19 and ST313 strains from Mali *in vivo*. CD-1 mice were orally infected and blood was collected at 3, 24, 72 and 168 h p.i. and bacterial loads were determined by performing viable counts. There were no significant differences between *Salmonella* Typhimurium ST19 and ST313 strains in bacterial loads of blood, spleen or liver at any of the time-points evaluated (Fig 1A, 1B and 1C). However, there was a trend towards higher bacteremia at 24 h in *S*. Typhimurium ST313-infected CD-1 mice.

When we tested the bacterial loads in the blood of BALB/c mice, we did not observe a significant difference in the bacterial counts between *Salmonella* Typhimurium ST19 and ST313 at 3 h p.i., however, a significant increase in bacterial load was observed for *Salmonella* Typhimurium ST313 (P < 0.001; Student’s t-test, two-tailed) at 24 h when compared to *Salmonella* Typhimurium ST19 (Fig 1D).

### Evaluation of clinical signs in rhesus macaques

Indian rhesus macaques were challenged perorally with either *Salmonella* Typhimurium I77 (ST19; n = 3) or *Salmonella* Typhimurium D65 (ST313; n = 3). Monkeys were monitored for diarrhea, dysentery, and lethargy. The ST19-infected animals had a significantly higher number of days with moderate-to-severe diarrhea than those infected with ST313 (P = 0.05, Mann-Whitney, one-tailed; Table 2). In addition, by using moderate-to-severe diarrhea as an endpoint, significantly more ST19-infected animals had diarrhea than those infected with ST313 (P = 0.05, Fisher’s exact test). By Day 10, all diarrhea had completely resolved. On days 3 and 4, lethargy and dysentery, respectively, were also observed in monkey DFK0 (infected with strain I77). Monkeys infected with *Salmonella* Typhimurium D65 presented with very mild diarrhea.

**Table 2:** Diarrhea, lethargy and dysentery elicited by *Salmonella* Typhimurium I77 (ST19) and D65 (ST313) in Indian rhesus macaques.

Strain	Animal ID	Day post-infection											Days with MSD^d^
		0	1	2	3	4	5	6	7	8	9	10	
I77 (ST19)	DFK0	-	-	-	+++ ^a^^,^ ^b^	+++^c^	+++	+++	++	+	+	-	5
	DFP4	-	-	-	+++	+++	-	-	-	-	-	-	2
	DG15	-	+	+	+++	+++	+++	+++	++	+	-	-	5
D65 (ST313)	DFN1	-	+	-	-	-	-	-	-	-	-	-	0
	DFT9	-	-	+	-	+	-	+	+	+	-	-	0
	DFV4	-	-	+	+	+	+	+	-	-	-	-	0

^a^ Severity of diarrhea.

+, mild (soft stool mixed with some watery stool);

++, moderate (watery stool);

+++, severe (watery mucoid stool).

^b^ Lethargy

^c^ Dysentery (blood observed in stool)

^d^ MSD, moderate-to-severe diarrhea

**Fig 2:**
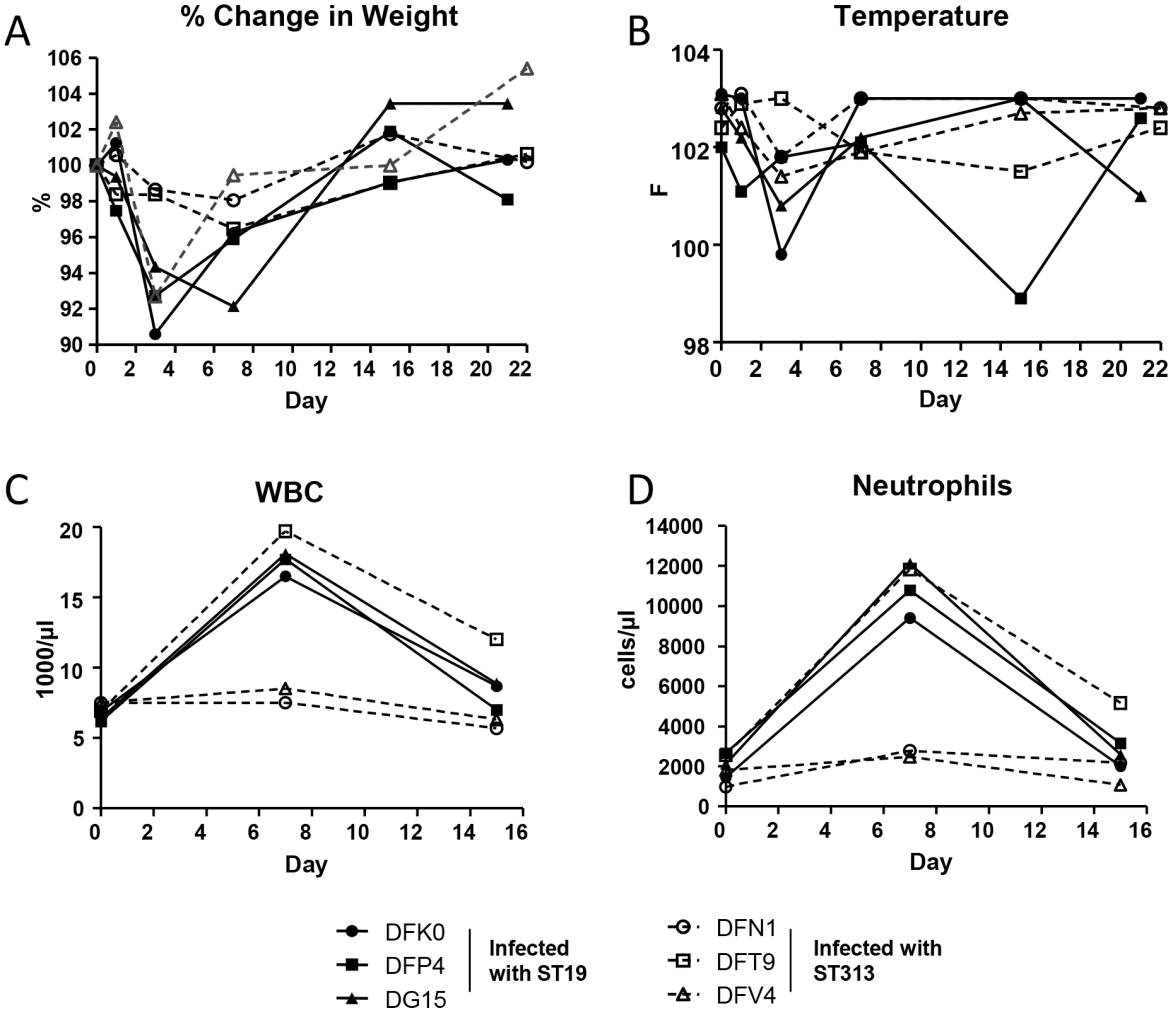
Clinical signs in rhesus macaques. Rhesus macaques infected with *Salmonella* Typhimurium I77 (ST19) or D65 (ST313) were measured for (A) Percent change in weight, and (B) Temperature (in Fahrenheit) on days 1, 3, 7, 15 and 21/22 days post infection. Blood from infected monkeys was collected on days 0, 7 and 15 and measured for (C) white blood cell (WBC) and (D) neutrophil counts.

The monkeys were monitored for changes in body temperature and body weight on Days 0, 1, 3, 7, 15 and 21 or 22. Close to 10% and 7% reduction in body weight was observed in two monkeys (DFK0 and DFP4) infected with *Salmonella* Typhimurium I77 (ST19) at 3 days p.i. (Fig 2A). At 7 days p.i., an 8% reduction in body weight was observed for DG15 which was infected with *Salmonella* Typhimurium I77 (ST19). Only one monkey (DFV4) infected with *Salmonella* Typhimurium D65 (ST313) showed weightloss, with a 7% reduction in body weight 3 days p.i. A similar pattern was observed for body temperature (Fig 2B). Most of the monkeys maintained their body temperature p.i. However, two monkeys infected with *Salmonella* Typhimurium I77 (ST19) had a mild reduction in body temperature at day 3 p.i.

Complete blood count analysis was performed on days 0, 7, and 15. Monkeys DFV4 and DFN1 infected with *Salmonella* Typhimurium D65 (ST313) had stable white blood cell (WBC) (Fig 2C) and neutrophil (Fig 2D) counts. Only one monkey (DFT9) infected with *Salmonella* Typhimurium D65 (ST313) showed an increase in WBC and neutrophil counts on day 7 p.i. This animal also had increased lymphocytic count (6240/μl) by day 15 p.i. when compared to its baseline lymphocytic count of 4002/μl on day 0 of the study (pre-infection). All three monkeys infected with *Salmonella* Typhimurium I77 (ST19) displayed a trend towards an increase in WBC and neutrophil counts on day 7 p.i. (P > 0.05; Fig 2C and 2D).

### Fecal shedding

**Fig 3:**
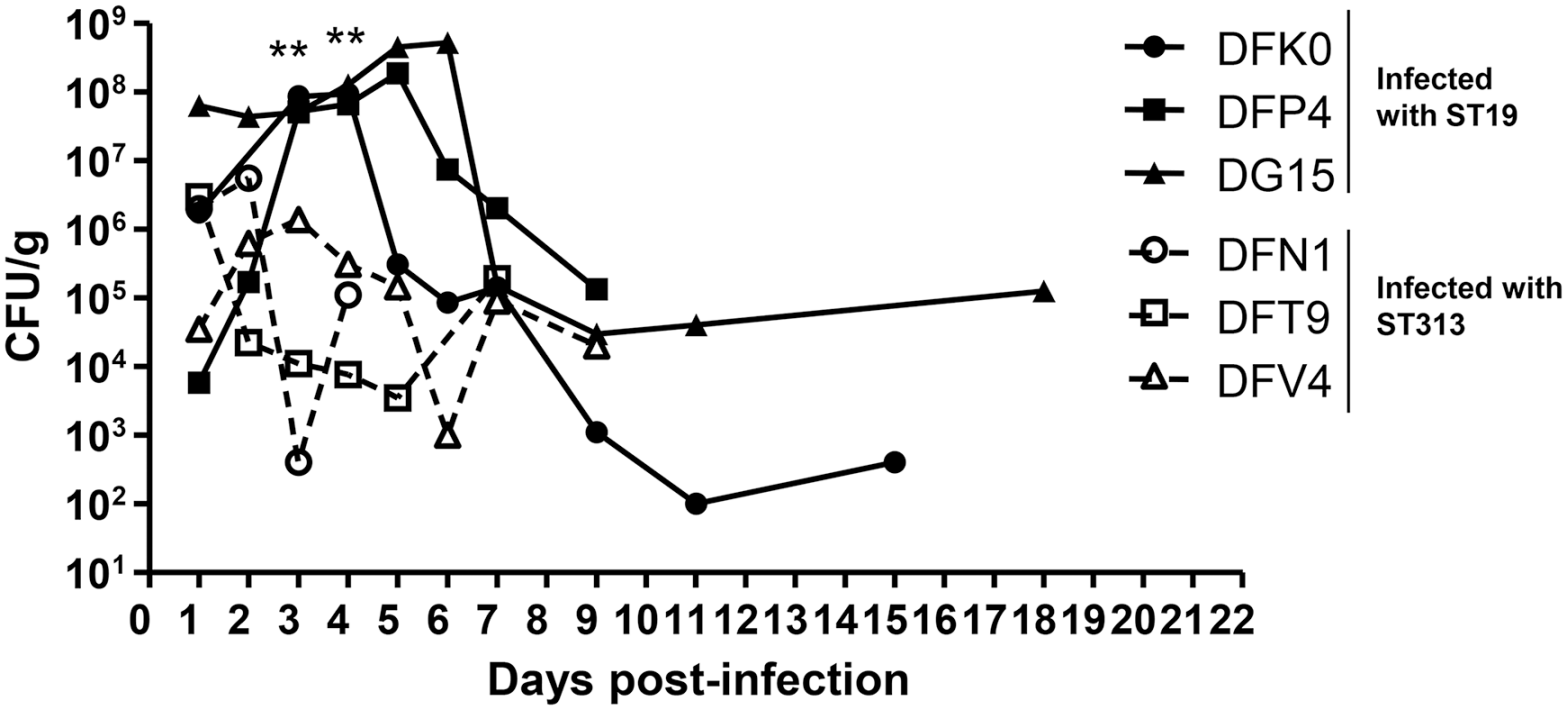
Shedding of *Salmonella* Typhimurium in the feces of rhesus macaques. Following infection with *Salmonella* Typhimurium I77 (ST19) or D65 (ST313), fecal samples were collected on days 1 to 7, 9, 11, 15, 18 and 21 or 22. Data is represented as CFU per gram of feces. ** represents P < 0.01, Student’s t-test, two-tailed.

Fecal samples from infected monkeys were collected on days 1 to 7, 9, 11, 15, 18 and 21 or 22. High levels of the bacteria were detected for both groups of monkeys. On days 1 and 2, there were no significant differences in bacterial load in the feces (P > 0.05) (Fig 3). However, on days 3 and 4 p.i., there was a significant increase in bacterial shedding in the feces of monkeys infected with *Salmonella* Typhimurium I77 (ST19), compared to monkeys infected with *Salmonella* Typhimurium D65 (ST313) (P = 0.006; Student’s t-test, two-tailed). All the monkeys that were infected with *Salmonella* Typhimurium D65 (ST313) stopped shedding bacteria by day 10 p.i. However, two monkeys infected with *Salmonella* Typhimurium I77 (ST19), continued to shed bacteria in the feces up to 18 days p.i.

### Qualitative and quantitative analysis of bacteria in tissues

Blood was collected on days 1, 3, 15 and 21 or 22 and tested by blood culture. One animal (DFT9) infected with *Salmonella* Typhimurium D65 (ST313) and another animal (DG15) infected with *Salmonella* Typhimurium I77 (ST19) had circulating bacteria in their blood on days 1 and 3, respectively, p.i. (Table 3).

**Table 3:** Qualitative and quantitative detection of *Salmonella* Typhimurium in blood, ileum, colon and mesenteric lymph nodes (MLN) of rhesus macaques.

		Detection of *Salmonella* Typhimurium in tissues at designated days post-infection						
Strain	Animal ID	Blood				Ileum	Colon	MLN^a^
		1	3	15	21/22	21/22 Swab (quantitative)	21/22 Swab (quantitative)	21/22 Swab (quantitative)
I77 (ST19)	DFK0	-^b^	-	-	-	-	-	- (4.0 x 10^2^ CFU/g)
	DFP4	-	-	-	-	- (1.9 x 10^3^ CFU/g)	- (5.7 x 10^4^ CFU/g)	-
	DG15	-	+	-	-	-	-	- (2.0 x 10^2^ CFU/g)
D65 (ST313)	DFN1	-	-	-	-	-	+^c^	-
	DFT9	+	-	-	-	-	-	-
	DFV4	-	-	-	-	-	-	-

^a^ MLN, mesenteric lymph nodes

^b^ + *Salmonella* spp. present following culture of swab or blood,—*Salmonella* negative following culture of swab or blood. No *Salmonella* were recovered from the spleen, liver, lung or axillary lymph nodes.

^c^ No bacteria detected using quantitative culture.

At euthanasia, qualitative culture from tissues was conducted by obtaining swabs of spleen, liver, lung, ileum, colon, MLN and axillary lymph nodes and the presence of *Salmonella* Typhimurium was detected by culture. *Salmonella* Typhimurium D65 (ST313) was detected in the colon of one animal (Table 3). Spleen, liver, ileum, colon and MLN were also collected from all the monkeys at euthanasia and quantitative bacterial counts were performed. One monkey (DFP4) infected with *Salmonella* Typhimurium I77 (ST19) had 1.9 x 10^3^ CFU/g of ileum and 5.7 x 10^4^ CFU/g of colon (Table 3). Two monkeys (DFK0 and DG15) infected with *Salmonella* Typhimurium I77 (ST19) also had 4 x 10^2^ CFU/g and 2 x 10^2^ CFU/g, respectively, isolated from their mesenteric lymph nodes.

### Histological evaluation of organ sections infected with *Salmonella* Typhimurium

**Fig 4:**
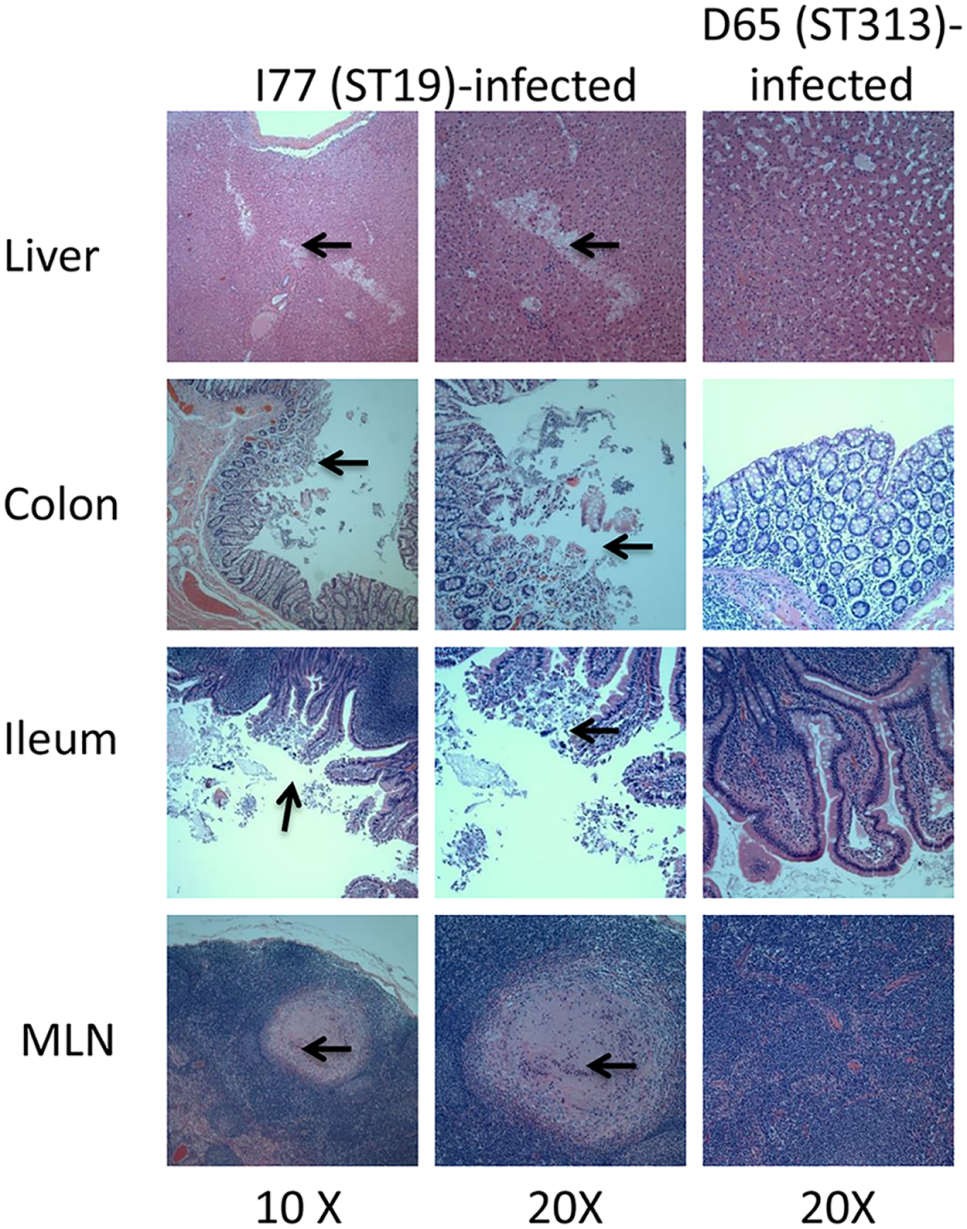
Histopathology of tissues infected with *Salmonella* Typhimurium. Hematoxylin and eosin-stained sections of liver, colon, ileum and mesenteric lymph node (MLN) from rhesus macaques. The images in the left (10X magnification) and middle (20X magnification) columns are from animals infected with *Salmonella* Typhimurium I77 (ST19). Images on the right (20X magnification) are from animals infected with *Salmonella* Typhimurium D65 (ST313). Areas of necrosis and lymphocytic infiltration of the liver section from a ST19-infected animal are indicated by arrows. Tissue sections from a ST19-infected animal show areas of erosion of mucosal epithelium of the colon and ileum combined with areas of infiltration of lymphocytes and plasma cells. These areas are indicated by arrows. Granulomatous areas consisting of multinucleated giant cells in the mesenteric lymph node of the animal infected with ST19 are indicated by arrows. All sections from D65-infected animals display essentially normal histology.

Hematoxylin and eosin-stained tissue sections of liver, spleen, colon, ileum and MLN from monkeys infected with either *Salmonella* Typhimurium I77 (ST19) or D65 (ST313) were analyzed for histopathology. Monkey DFK0 (infected with *Salmonella* Typhimurium I77 [ST19]) displayed pathological alterations in the liver, colon, ileum and MLN. Liver sections from this animal displayed multiple foci of lymphocytic infiltration and areas of necrosis (Fig 4). Focal necrosis and erosion of portions of the colonic epithelium was also evident. Areas of the colon displayed moderate multifocal lymphoplasmacytic colitis. The colonic mucosal epithelium was superficially eroded. The mucosal epithelium that was intact was lined by bacterial debris observed in the lumen and brush border of the colonic epithelium. Infiltration of a moderate number of lymphocytes, plasma cells and eosinophils in the lamina propria areas of the colon were evident. Areas of lymphoid hyperplasia were observed in the submucosal areas of the colon. Sections of the ileum from this animal showed moderately to severely blunted villi displaying moderate amounts of submucosal edema. The lamina propria displayed moderate numbers of lymphocytes, plasma cells and scattered eosinophils. The ileum displayed moderate multifocal lymphoplasmacytic and eosinophilic ileitis with blunting and lymphangiectasia. Multiple areas of granulomas composed of giant cells and eosinophils were seen in the MLN of this animal. The MLN changes indicated lymphoid hyperplasia and moderate granulomatous and eosinophilic lymphadenitis. Overall all animals infected with the *Salmonella* Typhimurium I77 strain (ST19) displayed varying degrees of pathology in the liver, ileum, colon and MLN. Monkeys belonging to the *Salmonella* Typhimurium D65 (ST313) infected group did not show any significant pathology in any of the organs evaluated (Fig 4).

## Discussion

In our present study, we evaluated the virulence of *Salmonella* Typhimurium ST19 and ST313 clinical strains in different animal models of infection. We first determined the i.p. LD_50_ of three *Salmonella* Typhimurium ST19 and ST313 strains in CD-1 mice and in adult and juvenile BALB/c mice (peroral infection). In all three models, our LD_50_ data suggests that the ST19 and ST313 genotypes are equally virulent in mice. These findings corroborate a recent study that showed that *Salmonella* Typhimurium ST313 isolates are not human host-restricted and instead produce an invasive phenotype in experimentally infected chickens [12]. When we infected BALB/c mice with *Salmonella* Typhimurium D65 (ST313) and I77 (ST19) we found significantly more ST313 bacteria in the blood at 24 h p.i. We hypothesize that this model of p.o. infection of BALB/c mice for 24 h could be used to examine the pathogenesis of ST313 strains and host responses to these bacteria.

We further tested the reference ST19 (SL1344) and ST313 (D23580) strains in BALB/c mice to determine differences in bacterial burden post peroral infection. In contrast to the results obtained by Yang et al [10], who showed a higher bacterial burden for D23580 (ST313) than SL1344 (ST19) in the spleen at day 3 and 5 p.i., we observed no difference in bacterial load between D23580 and SL1344 in the spleen, liver or blood (S2 Text and S1 Fig). Further work will need to be performed to determine if all the different iNTS models in mice are robust and can be repeated by various laboratories.

Interestingly, we also were not able to replicate the motility data reported by Yang et al [10] (S2 Text and S2 Fig). We have previously found that ST313 strains from Mali are less motile than ST19 strains [9]. Here, we repeated our analysis of I77 (ST19) and D65 (ST313) and also included some of the strains that were tested by Yang et al [10]; D23580, SL1344 and A130. We were able to replicate our previous findings whereby ST19 strains (SL1344 and I77) were more motile than ST313 strains (D65, D23580, A130 and 5579). In contrast, Yang et al [10] found that D23580 was more motile than SL1344, which in turn, was more motile than A130. Our data (previous and present) is supported by Carden et al [8] who showed that ST313 isolates express less *fliC* than ST19 isolates. Collectively, our data differs from Yang et al [10] in terms of *in vivo* virulence as well as motility. This could potentially be due to differences in the method of bacterial propagation or perhaps passaging of the bacteria has resulted in changes in the strains.

Our streptomycin model data was similar to Okoro et al [11] in that we did not observe a difference in bacterial load of the cecum (S2 Text and S3 Fig). However, unlike this previous study, we did not detect any differences in pathology.

Classical studies performed in the 1970’s showed that when 2–3 kg rhesus macaques were given 5 x 10^10^ CFU *Salmonella* Typhimurium, 80% of challenged animals exhibited diarrhea which lasted until 48–72 h p.i. [17,18,19]. We previously evaluated *Salmonella* Typhimurium I77 (ST19) and a candidate live-attenuated *Salmonella* Typhimurium vaccine in SIV-infected and SIV-uninfected Indian rhesus macaques with collaborators at the Vaccine Research Center, NIH [20] where we observed peak diarrhea 4–6 days p.i. in I77-infected animals. In the present study, we wanted to determine whether ST313 causes gastroenteritis using this model. For the first time, we have successfully shown that an ST313 strain does not elicit gastroenteritis in non-human primates. This data is supported by a previous study in which ST313 isolates were less able to induce fluid accumulation than ST19 isolates in bovine ligated loops [11]. Singletary et al [13] evaluated D23580 in rhesus macaque ligated ileal loops and found no difference in fluid accumulation compared to IR715 (ST19). They also saw no difference in inflammatory cytokine gene expression. Similarly, we saw no difference in proinflammatory cytokines circulating in sera of D65 and I77 infected rhesus macaques (S2 Text and S4 Fig). It is not clear why Singletary et al [13] did not observe any difference in fluid accumulation between ST19 and ST313. In this study, as well as our previous study [20], we used Indian rhesus macaques. Other groups have shown that the origin of non-human primates plays a role in their susceptibility to specific pathogens. A study conducted in rhesus macaques of Indian and Chinese origin displayed varying degrees of SIV pathogenesis in the two groups of animals [21]. Differential responses to *Shigella* infection have been reported in cynomolgus macaques of Chinese versus Mauritian origin [22]. If Singletary et al [13] used Chinese rhesus macaques, this may explain why their results differed from our own.

There were several limitations of our study. Firstly, we were not able to determine whether ST313 is indeed more invasive than ST19. Using blood culture, we detected I77 in one animal 3 days p.i. and D65 in one animal 1 day p.i. We believe that we were not able to detect bacteria in blood in additional animals due to the low volume tested by blood culture (3 ml) and also due to the limitations of blood culture itself which has low sensitivity [23]. We also were not able to evaluate bacteria in deep organs at early time-points as we wanted to monitor clinical signs and observe the entire progression of disease. We detected I77 in the ileum, colon and MLN at euthanasia (day 21 or 22) but only detected D65 in the colon of one animal. Now that we have established the model, we believe that additional experiments will allow us to determine whether ST313 is indeed more invasive than ST19. This could be achieved by examining bacterial burden in deep organs at early time-points (e.g., 3 days p.i.) and assessing inflammation by measuring pro-inflammatory cytokine production as well as pathological changes. Based on our *in vitro* data, we hypothesize that ST313 strains will be found in the blood and in deep organs (spleen and liver) at higher levels than ST19 bacteria; and will induce less pro-inflammatory cytokines.

Taken together, our data suggest that *Salmonella* Typhimurium ST313 isolates from sub-Saharan Africa are more invasive than ST19 isolates and do not elicit gastroenteritis. These findings support the clinical data whereby ST313 strains do not produce diarrhea in infants and children.

## Supporting information

S1 Text Supplementary methods. (PDF)

S2 Text Supplementary results. (PDF)

S1 Fig Bacterial loads in remote organs and blood. BALB/c mice were infected perorally with the reference *Salmonella* Typhimurium strains SL1344 (ST19) or D23580 (ST313). Bacterial loads were analyzed on days 3 and 5 in (A) spleen, (B) liver and (C) blood post-infection by determining the CFUs on Hy-Soy agar plates. Bacterial counts are presented as CFU per gram of tissue or CFU per milliliter of blood.
(PDF)

S2 Fig Motility assay. Swimming motility of two *Salmonella* Typhimurium ST19 (strains SL1344 and I77), and 4 ST313 (strains D65, D23580, A130 and 5579) were measured on motility agar plates (1% Tryptone, 0.5% NaCl, 0.4% agar). *** represents P < 0.001, Student’s t-test, two-tailed.
(PDF)

S3 Fig Colonization of the cecum. Streptomycin-treated mice were infected perorally with 5 x 10^8^ CFU of *Salmonella* Typhimurium I77, I41, S52 (ST19) or D65, Q55, S11 (ST313). On day 4 post-infection, the mice were euthanized and (A) the cecum were weighed and (B) bacterial burden in the cecum was determined by counting the CFU in the homogenized tissue.
(PDF)

S4 Fig Cytokine production in monkeys infected with *Salmonella* Typhimurium. Serum from rhesus macaques infected with *Salmonella* Typhimurium I77 (ST19) or D65 (ST313) were collected on days 0, 1, 3 and 15 and analyzed for pro-inflammatory cytokines IL-6 (A) and IFN-γ (B) using meso scale discovery analysis. Levels of IFN-γ were below detection for all days except day 1. The levels of cytokines are presented in pg per milliliter of sera.
(PDF)
