# Supplementary material for: Virulence of invasive Salmonella Typhimurium ST313 in animal models of infection
Source: PLoS Negl Trop Dis. 2017 Aug 4;11(8):e0005697. doi: 10.1371/journal.pntd.0005697 (PMC5559095; doi:10.1371/journal.pntd.0005697)
Supplement: S1 Text — (PDF) [file pntd.0005697.s001.pdf]

SUPPLEMENTARY DATA

Methods

Motility

The swimming motility of ST313 (D65, D23580, A130, 5579) and ST19 (SL1344 and I77) strains was determined by inoculating motility agar (1% tryptone, 0.5% NaCl, 0.3% agar) with bacteria and incubating at 37ºC for 14 hours. The zone of motility was measured. Data are expressed as the average from 17 independent experiments.

Bacterial load in mouse organs and blood

In another study, 8-week-old BALB/c mice were infected perorally with 5×10^8 CFU of either *Salmonella* Typhimurium SL1344 (n = 20) or D23580 (n = 20). Ten mice from each group were euthanized 3 days later and another 10 mice were euthanized on day 5. Blood, spleen and liver were harvested and bacterial loads were determined by viable counts.

Streptomycin-pretreated mouse model

Four hours prior to streptomycin treatment, food and water were removed. To remove normal flora, CD-1 mice were treated perorally with 20 mg of streptomycin (dissolved in 100 µl of sterile distilled H_2O). Food and water were returned to the cages and 24 h later, mice were infected perorally with 5×10^8 CFU of streptomycin-resistant *Salmonella* Typhimurium I77, I41, S52, SL1344 (ST19 strains) or *Salmonella* Typhimurium D65, Q55, S11 (ST313 strains). One group of mice was left uninfected and another received streptomycin alone. On day 4 p.i., mice were euthanized and the ceca were collected and weighed. The ceca were then homogenized and serially diluted to determine bacterial colonization by counting bacterial CFUs on HS media containing streptomycin.

Rhesus macaque procedures

(i) Animals

Indian-origin rhesus macaques (*Macaca mulatta*; age, 2 to 3 years old) were purchased from Alpha Genesis (Yamassee, SC). Animals were negative for macacine herpesvirus 1, SIV, simian retrovirus, simian T-lymphotrophic virus and *Campylobacter*, *Salmonella*, *Shigella*, and *Yersinia* spp. Prior to study initiation, all animals were quarantined for 3 months and tested negative for tuberculosis, and intestinal parasites. In addition, only animals that were negative for anti-*Salmonella* LPS serum IgG antibodies were used in this study. The study took place in the animal facility of the Program of Comparative Medicine (University of Maryland School of Medicine). The facility is AAALAC-international accredited, and all procedures in the study conformed to the policies and guidelines of the Institutional Animal Care and Use Committee of the University of Maryland School of Medicine. Animals were housed in a Biosafety Level 2 containment facility, and appropriate measures were taken to ensure safe handling practices while working with this pathogen. Animals were fed a commercial primate diet (Teklad 2050, Envigo, Indianapolis, IN) supplemented daily with fresh fruits and vegetables. In addition, animals were given supplemental food enrichment (fruit and nut mix, popcorn, peanuts, granola bars) once to twice weekly. All food items historically have been free of any bacterial contamination. Municipal drinking water (regularly tested in our facility and found free of any bacterial growth) was provided through an automatic watering system. In addition, a water bottle containing municipal drinking water was placed on each cage, to prevent interruption of water supply in the event of failure of the automatic watering system. All animals were housed individually (to enable individual collection of feces) in stainless steel primate caging for the duration of the study. Dehydration was treated with fluid therapy as needed. Pain was relieved using ketamine/buprenorphine at 0.005 – 0.03 mg/kg IM or SC every 12 hours until symptoms resolved. Animals were euthanized using an overdose of sodium pentobarbital. All procedures conformed to guidelines in the Guide for the Care and Use

of Laboratory Animals and the Animal Welfare Act and were fully compliant with recommendations in the Biosafety in Microbiological and Biomedical Laboratories Guide.

(ii) Intragastric inoculation

Animals were sedated with 10 mg/kg of ketamine administered intramuscularly (IM). After onset of sedation, an 8 to 14 Fr sized-orogastric tube (Tyco Healthcare Group LP Mansfield, MA) was passed through the mouth into the stomach of the animal. Location was verified by gently injecting 5 to 20 ml of air while auscultating the upper abdomen with a stethoscope to confirm gas sounds in the stomach. Following this, 10 milli equivalent (mEq) of 8.4% sodium bicarbonate (Neogen, Lexington, KY) was administered intragastrically through the tube. After waiting for 5 minutes, animals were inoculated intragastrically with 3 x 10^9 CFU of *Salmonella* Typhimurium suspended in 5 mEq of the sodium bicarbonate solution. After delivery of the inoculum, the orogastric tube was flushed with 5 - 10 ml of sterile saline to ensure delivery of the entire inoculum.

(iii) Blood collection

Blood samples were collected from animals at specific time intervals. Animals were sedated with Ketamine (10 mg/kg IM) prior to this procedure. Blood was collected via the femoral vein after ketamine sedation. Prior to blood collection, the area was aseptically prepared with a scrub of betadine followed by a rinse with 70% alcohol prior to venipuncture.

(iv) Clinical monitoring

Challenged animals were observed daily throughout the study for diarrhea, dysentery, fever, signs of respiratory illness, changes in food intake or any abnormal behavior. Rectal temperature using the Sure Temp Plus thermometer (Welch Allyn, New York) was monitored daily (after ketamine sedation). Monkeys that become ill (manifested by diarrhea, fever-body temperature exceeding 103 °F, dysentery or any other signs) were treated within 24 h of the time at which diarrhea or

dysentery was first observed. Such animals received fluid therapy (10 to 20 ml/kg Ringers Lactate Solution, intravenous injection given once or twice daily) as determined by a facility veterinarian.

(v) Blood chemistry, enzymes and differentials

Blood samples collected from animals at various time points of the study were analyzed by Antech Diagnostics (Lake Success, NY) for blood chemistry and complete blood counts. Blood chemistry measurements included total protein, globulin, albumin, albumin/globulin ratio, creatinine, urea, blood urea nitrogen, alkaline phosphatase, serum glutamic oxaloacetic transaminase (SGOT), serum glutamic pyruvic transaminase (SGPT), glucose, phosphorus, calcium, chloride, triglycerides and amylase. Complete blood counts included hemoglobin, hematocrit, white blood cells (WBC), red blood cells (RBC), mean corpuscular volume (MCV), mean corpuscular hemoglobin concentration (MCHC) and platelet counts. Differentials included lymphoctyes, eosinophils, monocytes, neutrophils and basophil counts.

(vi) Euthanasia and tissue collection

At the end of the experiment, animals were sedated with 10 mg/kg ketamine. Euthanasia was performed by administering sodium pentobarbital (100 mg/kg) intravenously. Blood and tissue samples (including spleen, axillary and mesenteric lymph nodes, liver, ileum, duodenum, colon, cecum) were collected at necropsy for histopathological and culture analysis.

(vii) Cytokine analysis

Cytokine analysis on serum samples was performed by using the Meso Scale Discovery (Rockville, MD) platform. Serum samples were assayed neat on a single custom U-PLEX Biomarker Group 1 (NHP) plate (K15068L-1), containing capture antibodies for IFNγ, IL-1β, IL-2, IL-4, IL-6, IL-8, IL-10, IL-12/23 p40, IL-17 and TNFα, as per the manufacturer's instructions. Individual samples were assayed in duplicate (on days 0 and 22) or triplicate (days 1, 3 and 15).
