# Supplementary material for: Virulence of invasive Salmonella Typhimurium ST313 in animal models of infection
Source: PLoS Negl Trop Dis. 2017 Aug 4;11(8):e0005697. doi: 10.1371/journal.pntd.0005697 (PMC5559095; doi:10.1371/journal.pntd.0005697)
Supplement: S2 Text — (PDF) [file pntd.0005697.s002.pdf]

SUPPLEMENTARY DATA

Results

Motility

ST19 strains (SL1344 and I77) were more motile than ST313 strains (D65, D23580, A130 and 5579) (S2 Fig).

Salmonella Typhimurium ST19 and ST313 persist at similar levels in remote organs

In a previous study by another group, it was reported that on day 3 post-infection, no differences in bacterial burden was observed in the gallbladder, however, on day 5 post-challenge they found a log 10 increase in the bacterial burden in mice infected with *Salmonella* Typhimurium D23580 (ST313) compared to the well-characterized reference strain SL1344 (ST19) [10]. The bacterial burden in the spleen of mice infected with *Salmonella* Typhimurium D23580 was significantly higher on both day 3 and day 5 post-challenge compared to mice infected with SL1344 [10]. In our current study, we also examined the bacterial burden of *Salmonella* Typhimurium D23580 (ST313) and SL1344 (ST19) in the spleen, liver, and blood. Our data contrasted the previously published report. We found no significant differences in the bacterial burden on day 3 or day 5 in the spleen, liver, and blood of mice infected with *Salmonella* Typhimurium D23580 or SL1344 (S1 Fig). In our hands, we were not able to recapitulate previous findings where D23580 was shown to penetrate to deep organs faster and to higher levels than SL1344.

Infection of *S*. Typhimurium ST19 and ST313 strains in a streptomycin-treatment model

To determine the ability of *Salmonella* Typhimurium ST19 and ST313 strains to colonize the cecum of CD-1 mice, we infected streptomycin-treated CD-1 mice with 3 ST19 (I77, I41 and S52) and 3 ST313 (D65, Q55, S11) strains and determined cecum weight and bacterial burden 4 days p.i. Overall, the cecum weights of mice infected with *Salmonella* Typhimurium ST19 or ST313 were lower than untreated mice or streptomycin-alone treated mice. There was no significant

difference in cecum weights of mice infected with either *Salmonella* Typhimurium ST19 or ST313 strains (S3A Fig). Furthermore, we observed a slight increase in bacterial burden in the cecum of mice infected with *Salmonella* Typhimurium ST19 strains compared to *Salmonella* Typhimurium ST313 strains; however this was not statistically significant. Mice infected with *Salmonella* Typhimurium SL1344 (the reference ST19 strain) had the lowest bacterial counts in the cecum 4 days p.i. (S3B Fig). Therefore, there was no significant trend observed towards greater colonization of the cecum between *Salmonella* Typhimurium ST19 and ST313 strains in this murine model of infection.

Cytokines in blood of Rhesus macaques

Serum samples obtained from non-human primates pre- and post-challenge were assessed for the presence of circulating cytokines. Several of the cytokines (IL-1β, IL-2, IL-4, IL-10, TNFα) were not identified in high concentrations at any time point. This was either due to the low sensitivity of the assays or low levels of circulating cytokines. Other cytokines showed no trend in either increase or decrease post-challenge (IL-8, IL-12/23p40, IL-17). Two cytokines (IL-6 and IFNγ) showed increased concentrations in sera at day 1 post-challenge (S4 Fig). IFNγ concentration decreased to pre-challenge levels by day 3, while IL-6 concentration was still elevated at day 3, but had subsided by the next time point at day 15. There was a good correlation between cytokine levels detected in individual monkeys, with the monkey with the greatest IL-6 levels also having the highest IFNγ levels. When comparing the two experimental groups, there was no discernible trend towards increased cytokine production associated with either bacterial strain.
