# Supplementary figures and images for: Virulence of invasive Salmonella Typhimurium ST313 in animal models of infection
Source: PLoS Negl Trop Dis. 2017 Aug 4;11(8):e0005697. doi: 10.1371/journal.pntd.0005697 (PMC5559095; doi:10.1371/journal.pntd.0005697)

S1 Fig

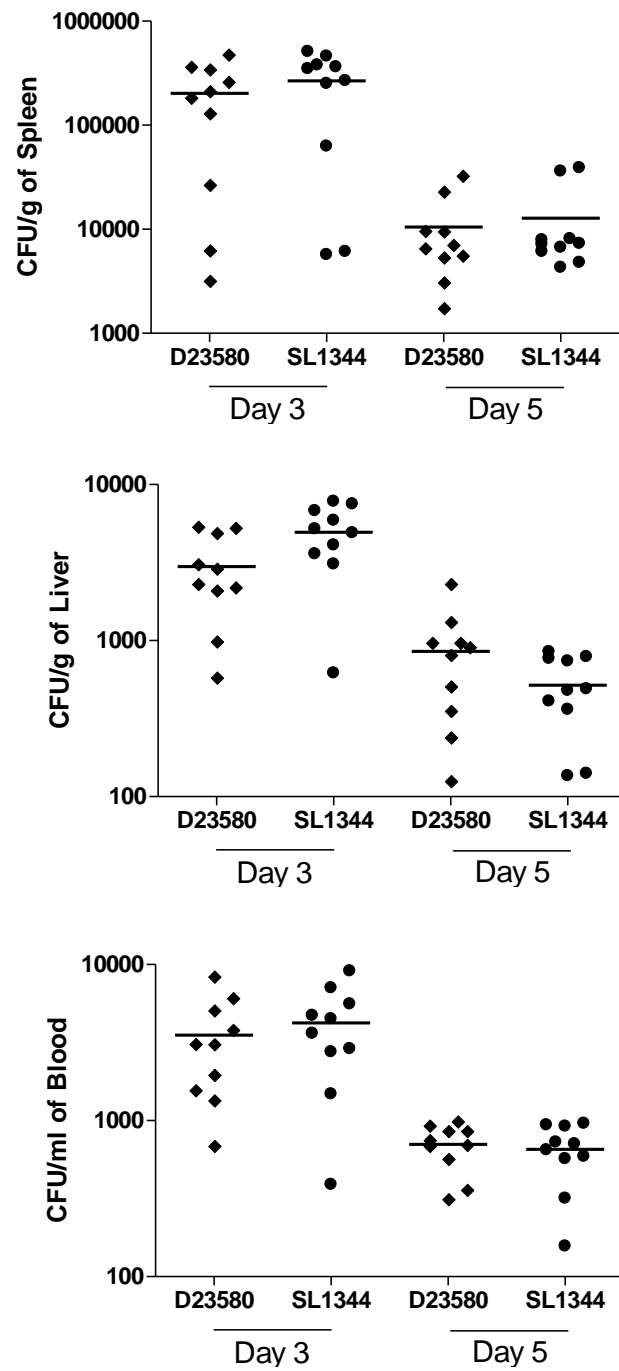

Supplement: S1 Fig — BALB/c mice were infected perorally with the reference Salmonella Typhimurium strains SL1344 (ST19) or D23580 (ST313). Bacterial loads were analyzed on days 3 and 5 in (A) spleen, (B) liver and (C) blood post-infection by determining the CFUs on Hy-Soy agar plates. Bacterial counts are presented as CFU per gram of tissue or CFU per milliliter of blood. (PDF) [file pntd.0005697.s003.pdf]

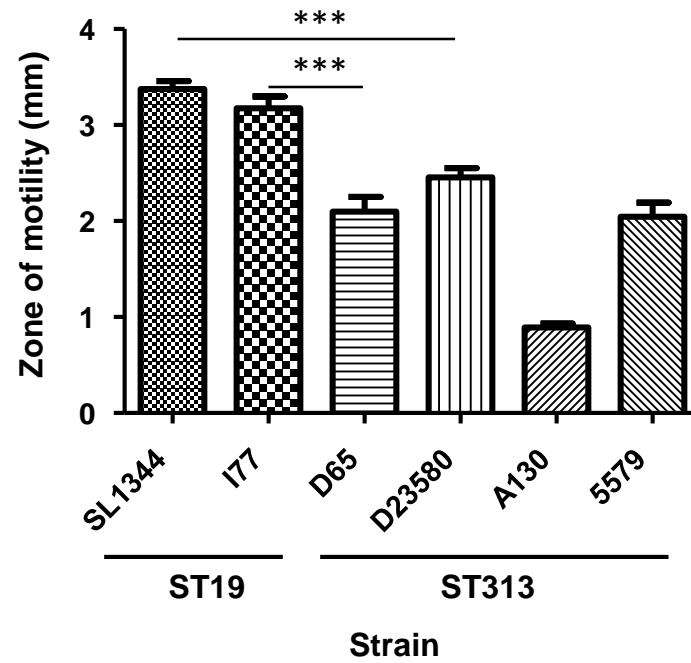

S2 Fig

Supplement: S2 Fig — Swimming motility of two Salmonella Typhimurium ST19 (strains SL1344 and I77), and 4 ST313 (strains D65, D23580, A130 and 5579) were measured on motility agar plates (1% Tryptone, 0.5% NaCl, 0.4% agar). *** represents P < 0.001, Student’s t-test, two-tailed. (PDF) [file pntd.0005697.s004.pdf]

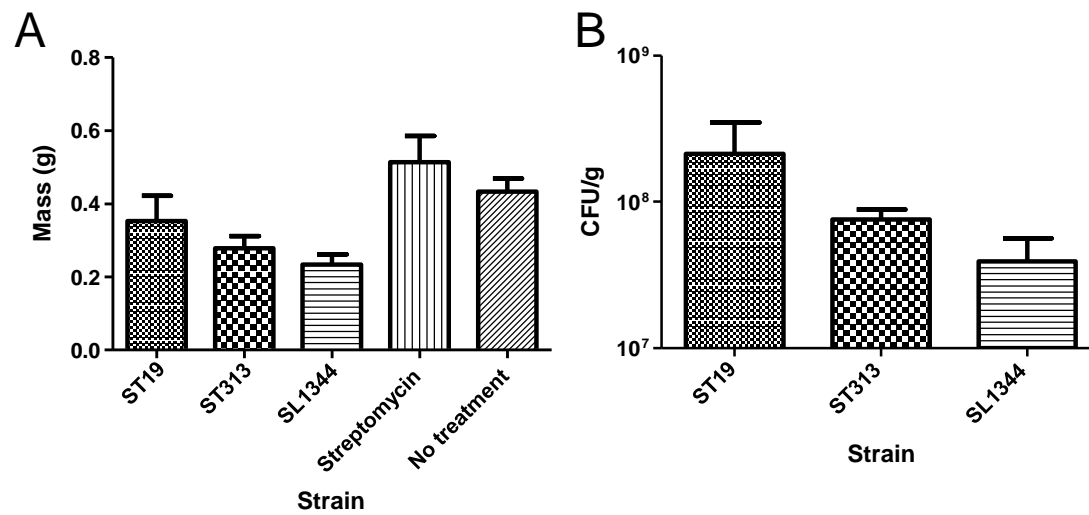

S3 Fig

Supplement: S3 Fig — Streptomycin-treated mice were infected perorally with 5 x 10^8^ CFU of Salmonella Typhimurium I77, I41, S52 (ST19) or D65, Q55, S11 (ST313). On day 4 post-infection, the mice were euthanized and (A) the cecum were weighed and (B) bacterial burden in the cecum was determined by counting the CFU in the homogenized tissue. (PDF) [file pntd.0005697.s005.pdf]

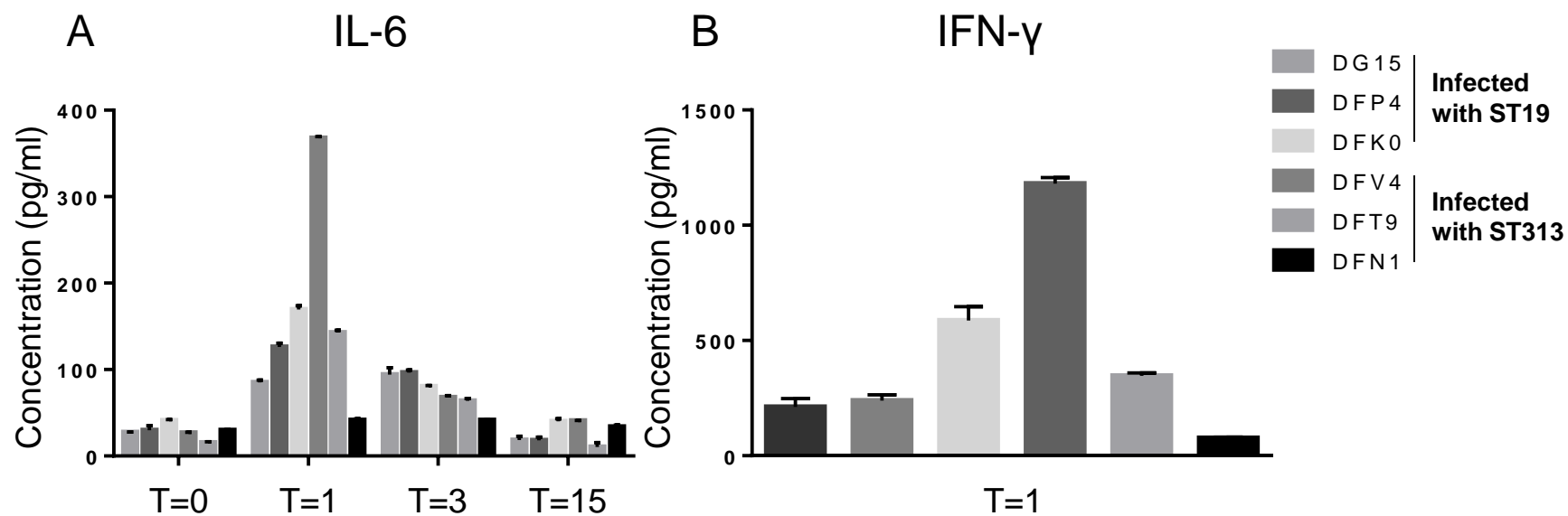

S4 Fig

Supplement: S4 Fig — Serum from rhesus macaques infected with Salmonella Typhimurium I77 (ST19) or D65 (ST313) were collected on days 0, 1, 3 and 15 and analyzed for pro-inflammatory cytokines IL-6 (A) and IFN-γ (B) using meso scale discovery analysis. Levels of IFN-γ were below detection for all days except day 1. The levels of cytokines are presented in pg per milliliter of sera. (PDF) [file pntd.0005697.s006.pdf]
